# Job vacancies at the European Centre for Disease Prevention and Control 

**DOI:** 10.2807/1560-7917.ES.2024.29.34.240822v

**Published:** 2024-08-22

**Authors:** Eurosurveillance editorial team

**Affiliations:** 1 European Centre for Disease Prevention and Control (ECDC), Stockholm, Sweden

The Centre has published the following **Temporary Agent** position:

- Expert Microbial Safety of Substances of Human Origin 

The application deadline expires at 12:00 noon on 26 September 2024 (Stockholm time).

For more information, please visit the ECDC website: https://erecruitment.ecdc.europa.eu/?page=advertisement

